# Trigeminal neuralgia caused by Dandy-walker malformation: A case report and systematic review of the literature

**DOI:** 10.1016/j.radcr.2021.07.049

**Published:** 2021-08-13

**Authors:** Juna Musa, Masum Rahman, Angela Guy, Ilir Ahmetgjekaj, Ali Guy, Ina Kola, Abu Bakar Siddik, Alireza Shoushtarizadaeh, Kristi Saliaj, Guri Hyseni, Fjolla Hyseni

**Affiliations:** a Department of Surgery, Mayo Clinic, Rochester, Minnesota; b Department of Neurologic Surgery, Mayo Clinic, Rochester, Minnesota; c Clinical Psychology Health Emphasis California School of Professional Psychology Alliant International University, Los Angeles, California; d Department of Radiology, UBT and UCC, Pristina, Kosovo; e Department of Physical Medicine and Rehabilitation, NY University, School of Medicine-NYU Medical Center, New York; f Department of Burns and Plastic Surgery, Tirana, Albania; g Department of Pain medicine, Mayo Clinic, Jackksonville, Florida; h Department of Neurologic Surgery, Mayo Clinic, Rochester, Minnesota; i Mother Teresa Hospital, Tirana, Albania; j Department of Pediatric Surgery, Hospital and University Clinical Service of Kosovo, Pristina, Kosovo; k NYU Langone Health, New York

## Abstract

Trigeminal neuralgia is a pain condition that affects the face along the distribution of the trigeminal nerve and can be recurrent and chronic. Dandy-Walker syndrome is a complex congenital brain anomaly that occurs during embryonic development of the cerebellum and the fourth ventricle. It is characterized by inferior cerebellar vermis hypoplasia and incomplete formation of the fourth ventricle. Dandy-Walker Syndrome is associated with comorbid genetic conditions. It can include congenital heart defects, eye abnormalities, intellectual disability, congenital tumors, and other brain defects such as agenesis of the corpus callosum. However, associations of trigeminal neuralgia and Dandy Walker syndrome have been an infrequent entity. Herein, we report a case of a 23-year-old female patient that presented with complaints of severe left orofacial pain over two years. MRI evaluation was consistent with Dandy-Walker malformation findings that we suspect caused the compression in the trigeminal root entry zone that ultimately gave rise to the patient's symptoms.

## Introduction

Dandy-Walker malformation (DWM) is a rare congenital CNS malformation that affects 1 in 25,000-35,000 live births each year [1]. It comprises the agenesis or hypoplasia of the cerebellar vermis, cystic dilation of the fourth ventricle and enlargement of the posterior fossa [2]. Hydrocephalus is evident in at least 90% of patients, most cases are diagnosed soon after birth [2]. As a result, children will most likely present with slowed motor development and elevated intracranial pressure, leading to irritability, lethargy, vomiting and in severe cases, altered levels of consciousness [3]. Jerky, uncoordinated movements and seizures may also be seen in children with DWS [3].

A number of associated conditions may coexist with DWS, including congenital heart defects, congenital tumors, and agenesis of the corpus callosum [2]. However, contributing factors include and are not limited to chromosomal defects, vertical transmission of certain viral infections, exposure to certain medications or toxins and maternal diabetes [4].

Diagnostic and therapeutic methods include prenatal screenings, MRI and ultrasound. Ventriculoperitoneal (VP) and cystoperitoneal (CP) shunts are performed in an attempt to provide surgical treatment of hydrocephalus and reduce the CSF buildup of fluids [5]. Early diagnosis and intervention are necessary to prolong life expectancy, improve symptoms associated with hydrocephalus and further reduce complications. The prognosis of DWS is variable, largely depending on theseverity of the symptoms and the presence associated malformations.

## Case report

A 23-year old female patient presented to the emergency room with complaints of sudden, severe left orofacial pain, mainly localized around her mouth. She had undergone a third molar extraction a few days prior to the ER visit. A temporomandibular joint (TMJ) subluxation during the dental procedure was initially suspected; therefore the patient was prescribed a course of NSAIDs. A week later the pain had resolved. However, the patient continued to experience recurrent episodes of acute severe left orofacial pain in the span of two years, for which she consulted several neurologists.

Further evaluation revealed severe paroxysmal pain in the distribution of the ophthalmic and maxillary branches of the trigeminal nerve (CN V). The rest of the neurological examination was within the normal limit, without any cranial nerve deficits and sensory disturbances. Past medical history is significant for Dandy Walker malformation (DWM). The patient received a diagnosis of trigeminal neuralgia and on the account of her underlying neurological condition, a MRI of the head was ordered as well.

**Fig. 1:**
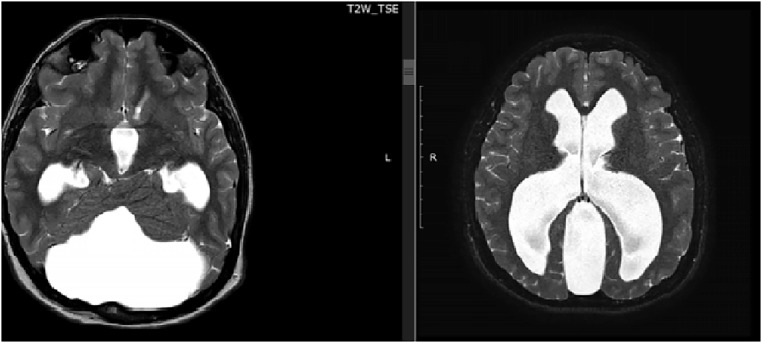
Axial T2 demonstrates an abnormal posterior fossa. The vermis is hypoplastic, below which a large cystic space continuous with the fourth ventricle is present. Prominent obstructive hydrocephalus is also evident.

**Fig. 2:**
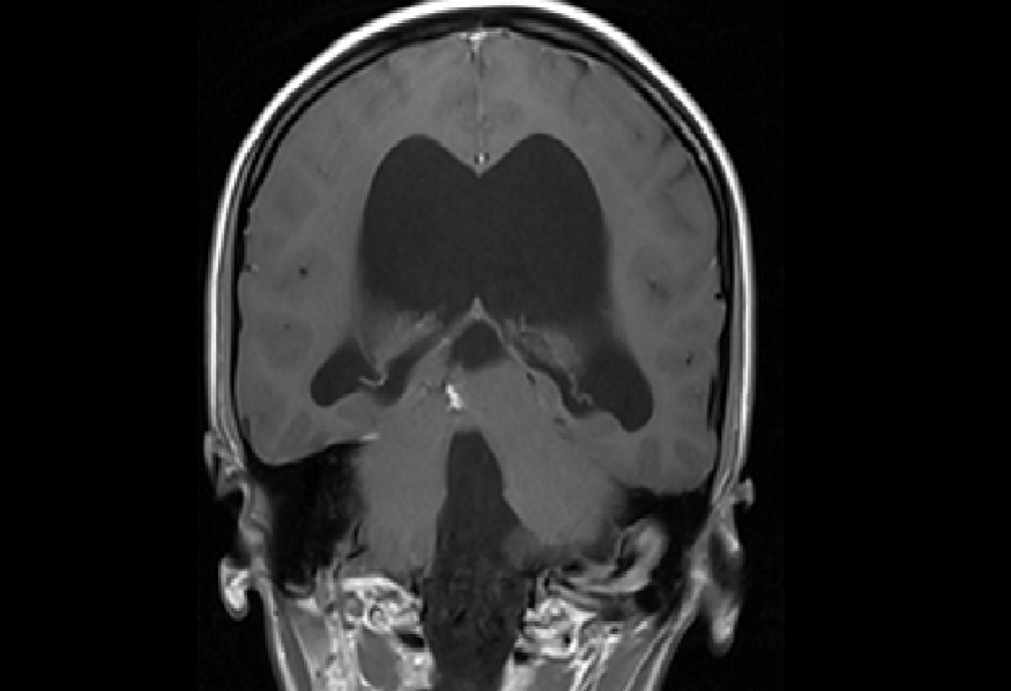
Sagittal T2 weighted image shows Dandy-Walker malformation, enlarged posterior fossa, and cystic dilation of the fourth ventricle image. Posterior fossa cystic abnormality communicating with the fourth ventricle is visible. Hydrocephalus is evident.

**Fig. 3:**
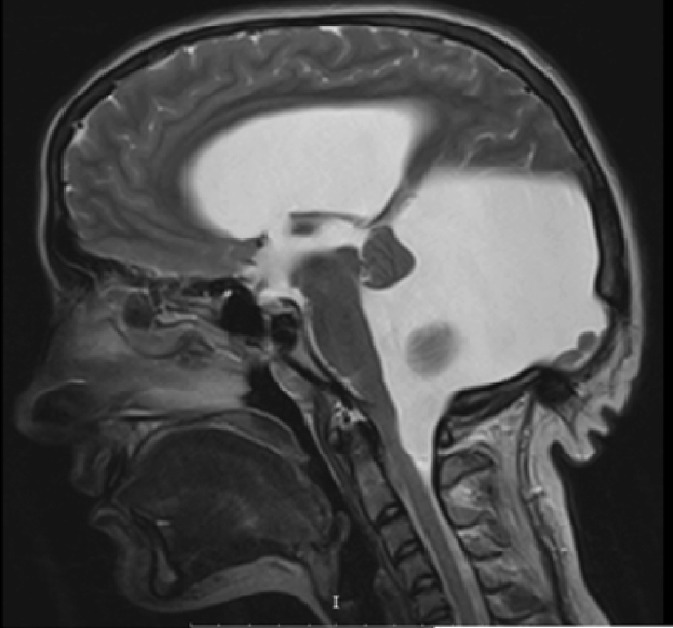
Coronal T1 weighted image shows posterior fossa cystic abnormality communicating with the fourth ventricle and massive hydrocephalus.

**Fig. 4:**
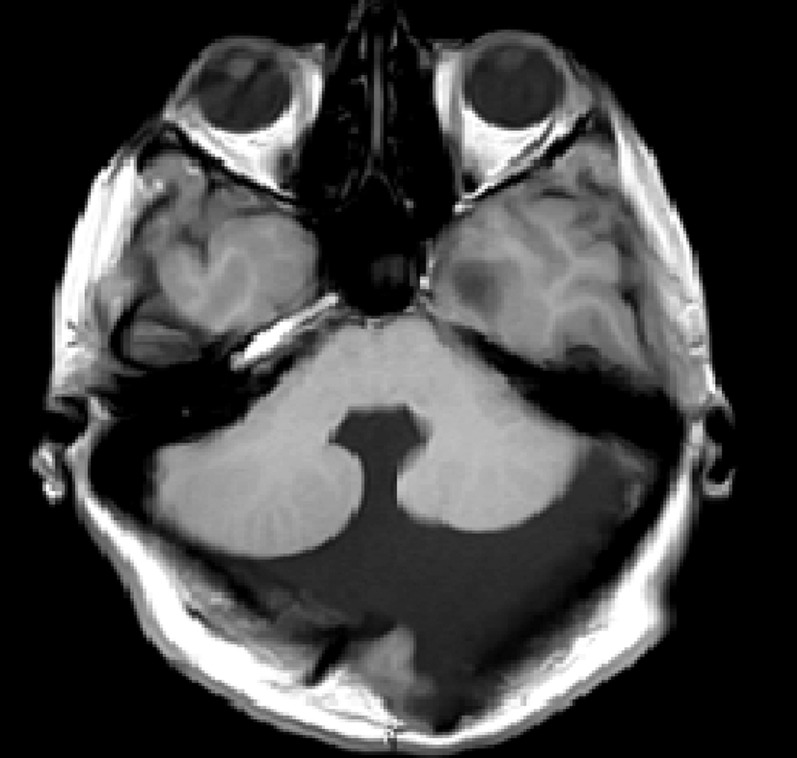
Axial T-1 weighted image shows a large cystic area that occupies the posterior fossa communicating directly with the fourth ventricle. Normal vermis cannot be identified, with a small vermian remnant appearing displaced and rotated superiorly. Features are consistent with Dandy Walker malformation with associated marked arrested hydrocephalus.

The MRI (Fig. 1, Fig. 2, Fig. 3–4) showed a hypoplastic cerebellar vermis, displaced and rotated superiorly, an enlarged cystic posterior fossa communicating with the fourth ventricle and the presence of hydrocephalus, consistent with Dandy Walker malformation, causing significant compression that hindered the proper visualization of the cerebellopontine angle cistern, displaced the Sylvian aqueduct as well as, we suspect, caused the compression in the trigeminal root entry zone that ultimately gave rise to the patient's symptoms.

A neurosurgical consult was ordered to discuss the possibility of a surgical treatment of the hydrocephalus through a shunt placement to relieve the compression. However, the patient opted for a more conservative approach. Thus, carbamazepine and diclofenac were prescribed for her symptoms and regular follow-up for the DWM.

## Discussion

Dandy Walker malformation is the most common congenital malformation affecting the posterior fossa and cerebellum, defined as the agenesis or hypoplasia of the cerebellar vermis, cystic dilatation of the fourth ventricle communicating with an enlarged posterior fossa causing an elevation of the tentorium, torcular Herophili, and lateral venous sinuses [10], [11], [12]. The most predominant clinical manifestation of the disorder is hydrocephalus, present in more than 90% of patients [10,13].

Most cases are sporadic and isolated, but DWM can also occur as a part of single-gene disorders, genetic syndromes, chromosomal abnormalities, Mendelian disorders, environmental factors including congenital infections or fetal alcohol exposure. [10,14] A few cases of de-novo mutations have also been reported [10]. DWM may be isolated or co-exist with other central nervous system anomalies such as complete or partial agenesis of the corpus callosum, occipital encephalocele, polymicrogyria, heterotopia or systemic malformations including cleft lip and palate, cardiac and neural tube defects [11].

Based on the heterogenous etiology several mechanisms have been proposed to explain the pathogenesis of Dandy Walker malformation. Two specific linked genes *ZIC1* and *ZIC4* have been implicated, they are ubiquitously expressed in the granular neuroprogenitor cells (GNPs) in the developing cerebellum and it has been suggested that mutations in these genes may disrupt the normal proliferation of GNPs, which in turn may be responsible for the vermian hypoplasia [15]. Another theory suggests DWM ought to be viewed in the context of a midline malformation syndrome rather than an isolated malformation [14]. Other studies suggest that DWM is caused by anomalies during the embryological development of the roof of the rhombencephalon. It has been argued that this may occur either due to an arrest in the development of the vermis, or a failure of fenestration of the fourth ventricle foramina promoting a consequent enlargement of the Blake's pouch, causing a compression of the cerebellar vermis that affects its normal development [12,16].

Trigeminal neuralgia is defined as sudden, brief, unilateral severe and recurrent episodes of pain limited to the distribution area of one or more branches of the trigeminal nerve, ophthalmic (V1), maxillary (V2) and mandibular (V3) [17,18]. It represents one of the most common causes of craniofacial pain [11]. The trigeminal nerve arises from three sensory and one motor nuclei. At the level of the pons, the sensory nuclei converge to give rise to the sensory root, whereas adjacent to the sensory root, the motor root originates from the motor nucleus. The trigeminal ganglion contains the neural bodies of the sensory fibres, with the sensory root emerging from the medial side of the ganglion, while the branches of the trigeminal nerve extend on its lateral surface. The exact pathogenesis of trigeminal neuralgia remains contentious, however the widely accepted NVC hypothesis suggests that pain is caused by neurovascular compression in the trigeminal root entry zone, usually by the superior cerebellar artery [18].

This sustained compression leads to demyelination and anomalies in the expression of voltage-gated sodium channels in the membrane [18]. These anomalies subsequently render afferent neurons hyperexcitable, prone to generating spontaneous nerve impulses and susceptible to cross excitation from adjacent axons, due to the demyelination [18], [19], [20], [21]. The synchronized afterdischarge activity, as well as the abnormal spread of action potentials between neurons are thought to be responsible for the paroxysmal pain episodes [18], [19], [20], [21].

**Table 1:** Table of content.

Cases	Age(years)	Sex	Symptom duration	Site of TN pain	MRI fingdings	Management	Symptomatic improvement
Ugur et al. [6]	32	M	15 years	Right	Cystic lesion occupied in the entire posterior fossa and hypoplastic vermis	Radiofrequency thermocoagulation rhizotomy (RF-TR)	Significant
Jha et al. [7]	65	F	3	Left	Ventriculomegaly with periventricular lucency	Micro- vascular decompression	Significant
Zhang et al. [8]	36	M	2 months	Left	Large posterior fossa cyst connecting the fourth ventricle resulting compression and distortion of the brainstem.	Conservative management with carbamazepine	NA

Associations between Dandy Walker malformation and trigeminal neuralgia are extremely rare. We believe the CSF build-up in the posterior fossa cyst accounted for the compression of the trigeminal nerve root, in the trigeminal root entry zone, within a few millimeters of entry at the level of the pons, that led to the symptomatology present in our patient.

To the best of our knowledge, Dandy Walker malformation as an etiological factor for trigeminal neuralgia has only been reported in three other separate occasions, by Ugur et al., Jha et al. and Zhang *et al.* [6], [7], [8].

## Literature Review

To date, only three reported cases have suggested an association between TN and DWM. Our case report represents the fourth case. When taking into account these three previously reported cases, as well as our patient, data shows that although DWM is a congenital malformation, and most patients have relevant neurologic manifestations since birth, the mean age of presentation with trigeminal neuralgia was 39 ± 18.16 years. The incidence of DWM by sex was reported 1.24 for males and 0.78 for females per 100,000 live births per year [9].

Although it is not conclusive, in our analysis, male-female distribution was equal, 1:1 (two males and two females). All of the reported cases had unilateral symptoms, although hypothetically, it was more likely for symptoms to be bilateral. Two out of four cases were managed conservatively with satisfactory outcomes. Due to the short patient cohort, it is not possible to conclude which is the better option. However, we postulate interventions for refractory cases.



## Conclusion

The findings in our case support the hypothesis that hydrocephalus due to the cystic enlargement of the posterior fossa, present in the Dandy Walker malformation, could compress the trigeminal nerve roots, that may lead to concurrent trigeminal neuralgia. There have been three previous cases that have reported the association of trigeminal neuralgia with Dandy-Walker malformation. However, a conclusive etiological relationship is yet to be proven. Our case report offers further support of this relationship.

## Authors declare

No conflict of interests.

## Patient consent

We have obtain the patient consent.
